# Predictors of functional outcome after extra-articular scapular fracture stabilization with Brodsky approach

**DOI:** 10.1007/s00590-025-04355-9

**Published:** 2025-06-19

**Authors:** Alejandro Mejia Bustamante, Ruben Dario Arias Perez, Laura Ximena Ramirez Carmona, Sebastian Calle Diaz

**Affiliations:** 1 Clínica Universitaria Bolivariana, Medellín, Colombia; 2 Pontifical Bolivarian University, Medellín, Colombia; 3 Pontifical Bolivarian University, Medellín, Colombia

**Keywords:** Scapula fracture, Shoulder injuries, Treatment outcome, Orthopedic procedures

## Abstract

**Purpose:**

Although surgical stabilization for displaced extra-articular scapular fractures is increasingly utilized, there remains limited evidence on predictors of functional recovery. This study aimed to evaluate long-term functional and radiographic outcomes after stabilization using the Brodsky posterior approach and to determine whether glenopolar angle or lateral border medialization independently predicts postoperative function. We hypothesized that improved radiographic parameters would be associated with better outcomes.

**Methods:**

This retrospective cohort included 16 patients with displaced extra-articular scapular fractures treated with open reduction and internal fixation via the Brodsky approach. Functional recovery was assessed using the Constant score at 48 months postoperatively. Pre- and postoperative glenopolar angle and lateral border medialization were measured. Paired comparisons and multiple linear regression were used to identify predictors of outcome.

**Results:**

All patients achieved full fracture union without complications or need for reoperation. The median Constant score of the operated shoulder was 94 (IQR 88–95), compared to 96 (IQR 93–96) in the contralateral shoulder (*p* = 0.002). Despite statistical significance, the 2-point difference did not exceed the minimal clinically important difference for shoulder function. Postoperative improvements in glenopolar angle and medialization were significant. The contralateral Constant score was the only independent predictor of postoperative outcome (*β* = 1.52, *p* = 0.001). Radiographic variables were not predictive.

**Conclusion:**

The Brodsky approach yields excellent long-term outcomes. Although radiographic correction is achieved, the preoperative function of the contralateral shoulder remains the most reliable indicator of postoperative recovery potential.

**Supplementary Information:**

The online version contains supplementary material available at 10.1007/s00590-025-04355-9.

## Introduction

Scapular fractures are rare, comprising less than 1% of all fractures and 3–5% of shoulder girdle fractures [1]. They occur primarily in men, particularly those between 40 and 60 years of age. Although these fractures can result from different trauma mechanisms, high-energy injuries such as motor vehicle accidents or major falls account for about 22% of cases and are often accompanied by other fractures [1]. The most associated fractures involve the clavicle (9%) and the proximal humerus (5%), alongside significant thoracic injuries like lung contusion and hemothorax [1, 2].

The most common types of scapular fractures involve the scapular body (52%) and the glenoid fossa (29%) [3]. Despite their complexity, nonoperative treatment is the preferred approach in 80% of cases. However, for certain fracture patterns, particularly those affecting glenoid or severely displaced fractures, up to 33% of patients require surgical intervention [4]. Given their frequent association with severe injuries and the potential for delayed diagnosis, early recognition and appropriate management are essential to optimizing patient outcomes [2].

Historically, scapula fractures have been treated non-surgically due to the challenging surgical approach posed by the bone’s deep anatomical position and extensive muscular attachments [5]. Several factors contribute to this trend, including the belief that the glenohumeral joint’s wide range of motion compensates for scapular malalignment, the prioritization of life-threatening injuries in polytrauma patients, and the relative inexperience of many surgeons with scapular fracture fixation [6]. While most scapular body fractures heal with conservative treatment, severe or progressive displacement can lead to functional limitations [7, 8]. Advances in imaging and a better understanding of shoulder biomechanics have prompted a reconsideration of surgical indications, particularly for cases involving significant displacement [8]. Notably, post-injury pain tends to be higher in nonoperatively treated fractures of the scapular body, neck, and glenoid, emphasizing the need for individualized treatment strategies to optimize recovery [7].

Although surgical indications for scapula fractures remain a topic of debate, certain criteria are commonly considered. Scapular body fractures may warrant surgical intervention if medialization of the lateral border exceeds 20 mm, angular displacement is greater than 45°, or if there is a combination of medialization over 15 mm with angulation beyond 30°. Additional factors include double disruption of the superior shoulder suspensory complex with displacement over 10 mm, a glenopolar angle less than 20°, or an open scapular fracture. Scapular neck fractures share many of these criteria but are typically considered for surgery when displacement exceeds 1 cm or angular displacement is greater than 40° [6, 7, 9, 10]. Despite these proposed guidelines, no universal consensus has been established, and surgical decision-making increasingly takes biomechanical dysfunction into account to optimize patient outcomes [5].

The posterior approach described by Judet remains the most used technique for scapular fractures. However, concerns about infraspinatus atrophy and external rotator weakness have driven the development of alternative methods. In response, various modifications of the original approach and minimally invasive techniques have been introduced to improve outcomes. Recently, there has been a growing focus on reducing soft tissue trauma, leading to a shift toward less invasive procedures that minimize muscle and tendon detachment while still ensuring adequate fracture stabilization and exposure [9, 11–13].

Among these alternatives, the Brodsky posterior approach has gained attention for its ability to preserve the posterior deltoid and minimize external rotator dysfunction by utilizing the interval between the infraspinatus and teres minor [14]. This technique is particularly useful for lateral pillar fractures of the scapula, and with a proximal extension of the standard longitudinal incision, it allows for secure acromion fixation while also permitting posterior glenohumeral capsule opening for improved visualization of intra-articular fracture lines involving the glenoid fossa. Compared to traditional approaches, Brodsky’s technique further reduces soft tissue trauma and muscle detachment, contributing to excellent functional outcomes [15].

This study aims to present the results with a modified posterior approach for scapular fractures, proposed by Brodsky et al. [14], at a high-volume trauma referral center. We evaluate the functional outcomes, complication rates, and overall surgical effectiveness, with a follow-up period of up to 48 months.

## Materials and methods

### Study design and patient selection

This study presents a retrospective cohort of patients with extra-articular scapular fractures meeting surgical criteria for surgery who were treated at a high-volume trauma referral center from January 2018 to January 2023. All procedures were performed using the Modified Posterior Brodsky Approach by a fellowship-trained shoulder orthopedic surgeon.

The study included patients over 18 years old with extra-articular scapular fractures that met the indications for open reduction and internal fixation (ORIF). Patients with intra-articular fractures or those with a history of prior scapular ORIF were excluded. A non-probabilistic, consecutive sampling method was used, in which all eligible patients treated during the study period were included without randomization. A total of 20 patients were initially considered for this study; one was excluded based on the inclusion and exclusion criteria, and three were lost to follow-up. Finally, 16 patients met adequate follow-up and inclusion–exclusion criteria.

### Radiological and clinical assessment

All patients underwent standardized imaging that included anteroposterior (AP), axillary, and scapular Y-view radiographs, complemented by computed tomography (CT) for accurate fracture characterization and surgical planning. Radiographic parameters analyzed were the glenopolar angle and the medial displacement of the lateral scapular border, both pre-and postoperatively. Fracture healing time was not specifically recorded. Consolidation was assessed through standard radiographs during follow-up. CT scans were not used for healing assessment.

Functional assessment was performed using the Constant score at 48 months postoperatively, with the contralateral (uninjured) shoulder serving as a control for comparison. Clinical evaluation included the assessment of shoulder range of motion, encompassing forward flexion, lateral elevation, internal rotation, and external rotation. These measurements were documented as part of the Constant score form, which was the primary functional assessment tool used. External rotation was evaluated through the five standard active movements outlined in the Constant score (hand behind head, hand on head, hand to mouth, hand behind back, and hand to lumbar spine). Internal rotation was recorded based on the highest anatomical position the patient could reach behind the back (e.g., Sacroiliac joint, L5, T12). This approach ensured standardized and reproducible documentation of functional shoulder mobility across all patients.

Fractures were classified according to the AO Foundation/Orthopaedic Trauma Association (AO/OTA) system. Demographic data (age, sex, limb laterality) and trauma mechanism (high- or low-energy) were documented. Associated injuries (e.g., clavicle, ribs, pelvis, thoracic trauma) were recorded systematically. Complications monitored included infection, neurovascular injury, fixation loss, and hardware failure. All evaluations were performed by fellowship-trained orthopedic trauma surgeons using a standardized protocol.

### Management of associated injuries

Associated orthopedic injuries were managed based on anatomical location, severity, and overall clinical status. In cases where the additional injury involved the ipsilateral shoulder girdle—such as clavicle or proximal humerus fractures—surgical fixation was performed during the same operative session as the scapular procedure. Conversely, when associated injuries required surgical intervention in other anatomical regions (e.g., pelvic ring, distal radius, or elbow), they were treated in a staged manner during separate procedures. Injuries that did not meet operative criteria were managed conservatively following institutional trauma protocols.

### Postoperative rehabilitation

All patients followed a standardized rehabilitation protocol under the supervision of the institutional physical therapy team. The program began with immobilization using a sling for 2 weeks, followed by the initiation of passive- and active-assisted range-of-motion exercises starting in the third week. Progressive strengthening exercises were introduced after 6 weeks, depending on each patient’s functional recovery.

Patients with isolated scapular fractures generally followed this timeline without modification. In contrast, for those with associated orthopedic injuries, such as clavicle or rib fractures, rehabilitation milestones were adjusted individually based on pain levels, fracture consolidation, and the need for additional surgical procedures. These associated injuries were managed either conservatively or with staged operative interventions, according to clinical severity and stability. Despite these individual adaptations, all patients adhered to the same general rehabilitation framework, ensuring consistency in therapeutic objectives across the cohort.

### Statistical analysis

For statistical analysis and data visualization, Python (version 3.11) was used, employing the SciPy library for statistical testing, Pandas for data handling, and Seaborn and Matplotlib for graphical representation. Normality was assessed using the D’Agostino and Pearson omnibus test. Since most variables did not follow a normal distribution, results were primarily reported as medians with interquartile ranges (IQR). However, for variables that demonstrated a normal distribution, data were presented as means with standard deviations (±). Accordingly, nonparametric tests were applied: the Wilcoxon signed-rank test was used for paired comparisons, and the Mann–Whitney U test was used for unpaired data. Spearman’s rank correlation coefficient was used to evaluate associations between continuous variables. A two-tailed *p* value < 0.05 was considered statistically significant throughout the analysis.

Additionally, a multiple linear regression analysis was performed to identify predictors of functional outcomes measured by the Constant score at 48 months postoperatively. Independent variables included pre- and postoperative glenopolar angles, pre-and postoperative medialization, patient age at surgery, and the Constant score of the contralateral shoulder. Cases with missing data were excluded from the model. Linearity, normal distribution of residuals, and multicollinearity were assessed before model fitting. The model was fitted using the ordinary least squares method.

### Surgical technique

**Fig. 1:**
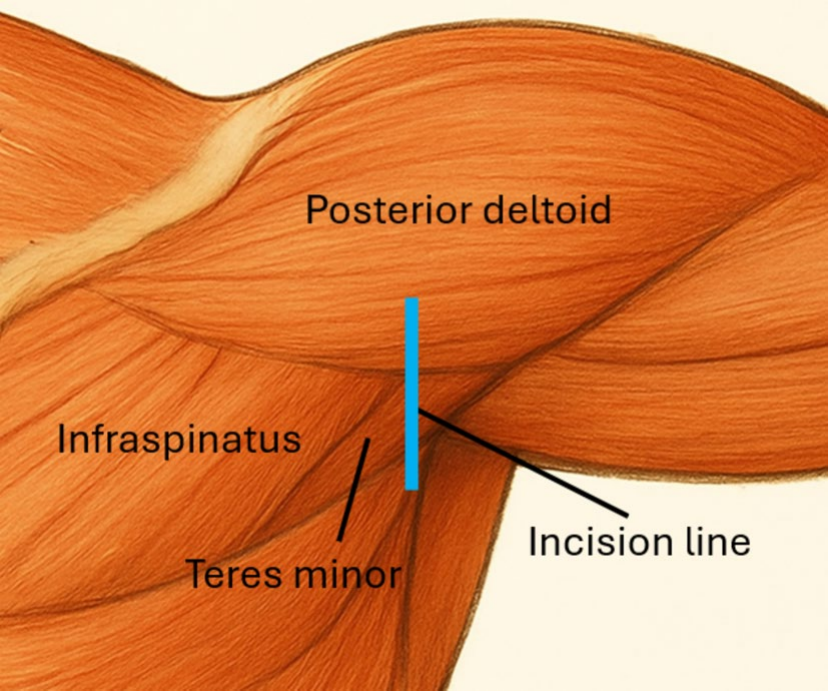
Representative illustration of the surgical incision. The blue line indicates the posterior skin incision used in the Brodsky approach, located between the posterior deltoid and the infraspinatus/teres minor interval. The illustration is author-generated

**Fig. 2:**
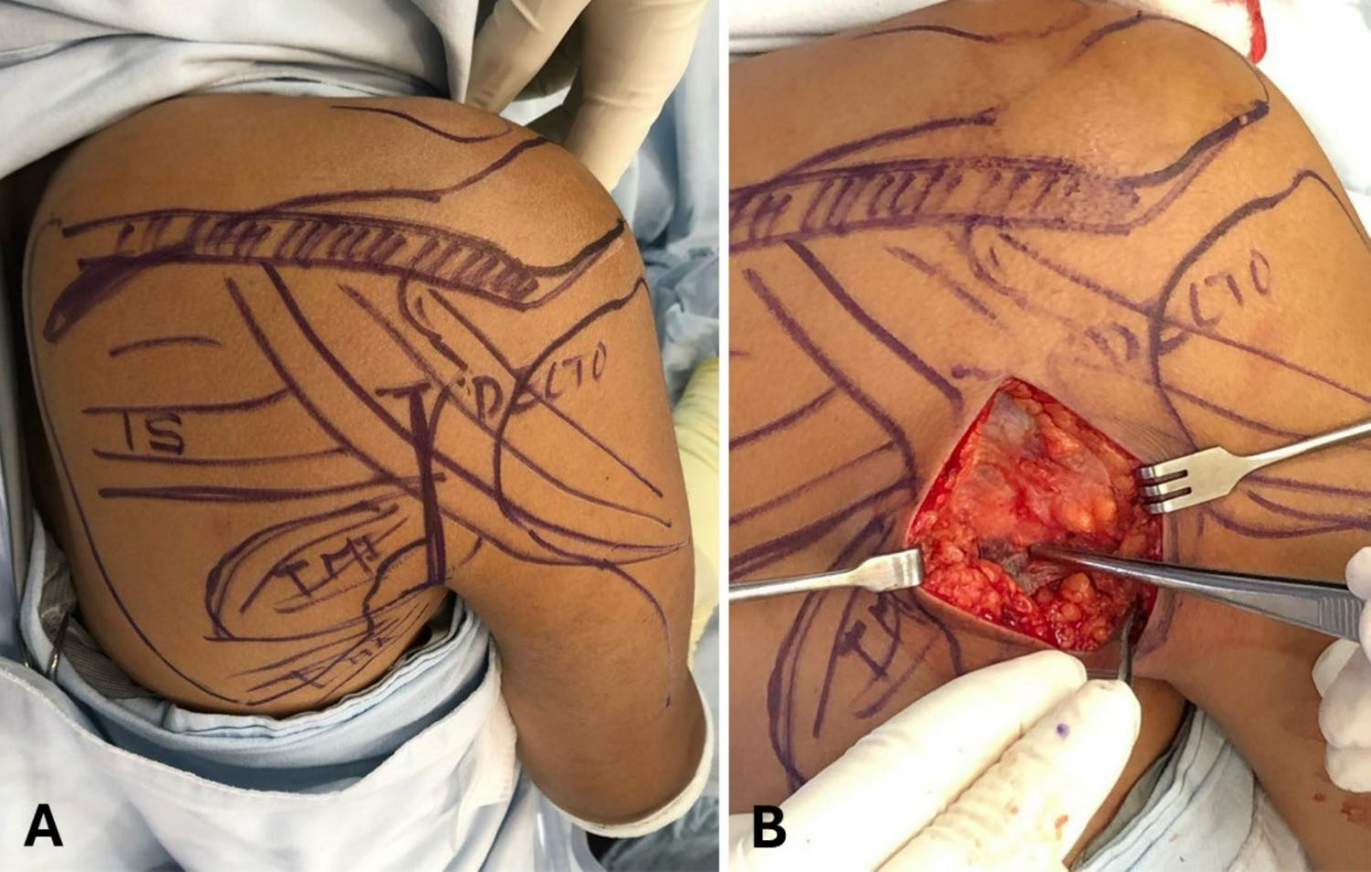
Incision and superficial dissection. **A** The main anatomical structures and the incision line are marked. **B** After completing the approach, the muscular fascia covering the deltoid and teres minor muscles are identified

All procedures were performed with the patient positioned in the lateral decubitus position, slightly leaning forward to optimize exposure of the scapula. The affected upper limb was prepped and draped freely to allow full mobility during the operation. The main anatomical structures are marked before the incision. A straight, vertical incision was made approximately 4 cm posterior to the acromion, in line with the posterior glenohumeral joint axis, and extended caudally for around 6–8 cm (Figs. 1, 2).

**Fig. 3:**
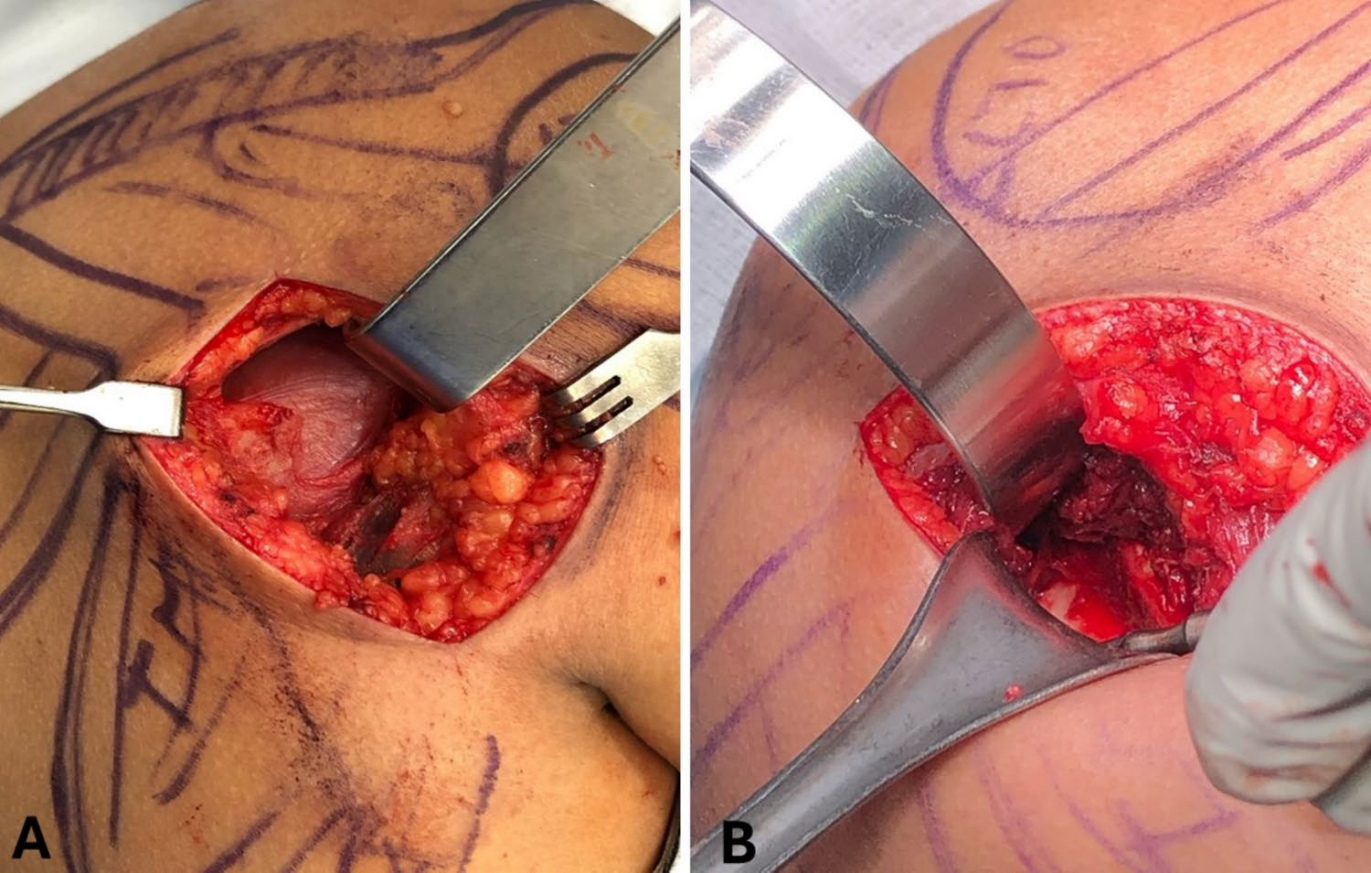
Deep dissection between the teres minor and the infraspinatus. **A** The deltoid is elevated and mobilized superiorly, allowing observation of the plane between the infraspinatus and teres minor muscles. **B** The intermuscular plane is approached, and the lateral border of the scapula is exposed

After superficial dissection, the posterior border of the deltoid muscle was identified (Fig. 2). Without detachment, the posterior deltoid was gently elevated and mobilized superiorly (Fig. 3). Additional exposure, if required, could be achieved by releasing a small portion, up to 2 cm, of its medial attachment along the scapular spine. To enhance deltoid mobility, the arm was positioned in approximately 90° of abduction and neutral rotation.

A natural interval between the infraspinatus and teres minor was identified and developed, following the deep fascial plane of the infraspinatus (Fig. 3). This allowed direct access to the lateral scapular border, the neck, and posterior glenoid rim. The infraspinatus was retracted superiorly, and the teres minor inferiorly, ensuring minimal traction to preserve the suprascapular nerve as it exits through the spinoglenoid notch.

At the level of the lateral scapular border, roughly 5–6 cm inferior to the glenoid, the ascending branch of the circumflex scapular artery was commonly encountered and ligated. Fracture fragments were visualized, mobilized, and anatomically reduced under direct vision. Internal fixation was performed using one or two plates per case, depending on fracture complexity and fragment distribution. Both 3.5-mm and 2.7-mm non-locking and locking plates were used. All implants were contoured intraoperatively to match the scapular anatomy; no pre-contoured anatomical plates or patient-specific implants were employed. Plate size and configuration were selected based on fragment size, anatomic location, and material availability within the institution. Closure was completed in layers, respecting fascial planes and minimizing soft tissue trauma.

## Results

A total of 16 patients who underwent open reduction and internal fixation (ORIF) of extra-articular scapular fractures were evaluated with a mean postoperative follow-up of 50 months (± 17.1). The mean age of the cohort was 38.2 years (± 9.7). The 81.2% (13 out of 16) of patients were male. Most injuries involved the non-dominant upper limb (87.5%), and the left shoulder was affected in 56.2% of cases. All patients sustained high-energy trauma, such as traffic collisions or occupational accidents. The mean time from trauma to surgery was 7.6 days (± 3.6).

Additionally, a significant proportion (75%) of patients presented with concomitant injuries, including fractures of the clavicle, ribs, pelvis, distal radius, and facial bones, as well as thoracic injuries like hemothorax and pneumothorax. Only four patients had isolated scapular fractures without associated injuries. Among the 12 patients with additional injuries, 10 (83.3%) required surgical management, either simultaneously with the scapular fixation or in staged procedures, depending on the anatomical location and clinical stability (Supplementary Table 1).

**Fig. 4:**
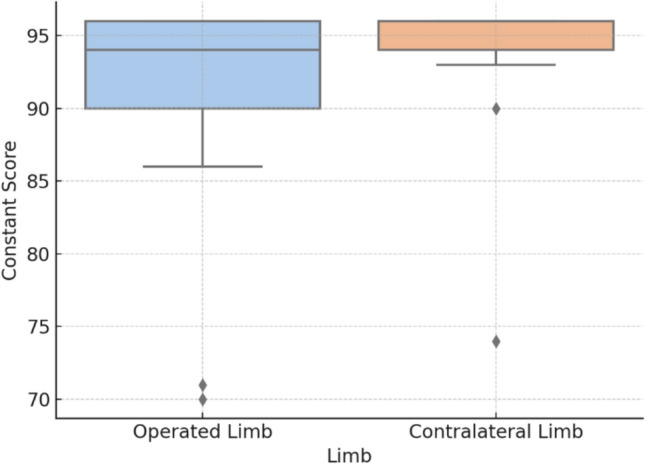
Comparison of the results of the Constant score. Boxplot comparing Constant scores between operated and contralateral shoulders at 48 months. A statistically significant difference was observed (*p* = 0.002)

Regarding functional outcomes, the Constant score for the operated shoulder had a median of 94 points (IQR 88–95). The contralateral shoulder demonstrated a higher median Constant score of 96 points (IQR 93–96). There is a statistically significant difference between these values with a median difference: 2 points, 95% CI 0–5; *p* = 0.0020). Figure 4 shows the comparison of the Constant score between the affected and contralateral limb or shoulder. At the final follow-up, the median active forward flexion was 158°, and lateral elevation was 160°. External rotation was most commonly graded as 5/5 active movements based on the Constant score criteria. Internal rotation typically reached the T7 vertebral level (Supplementary Table 1).

**Fig. 5:**
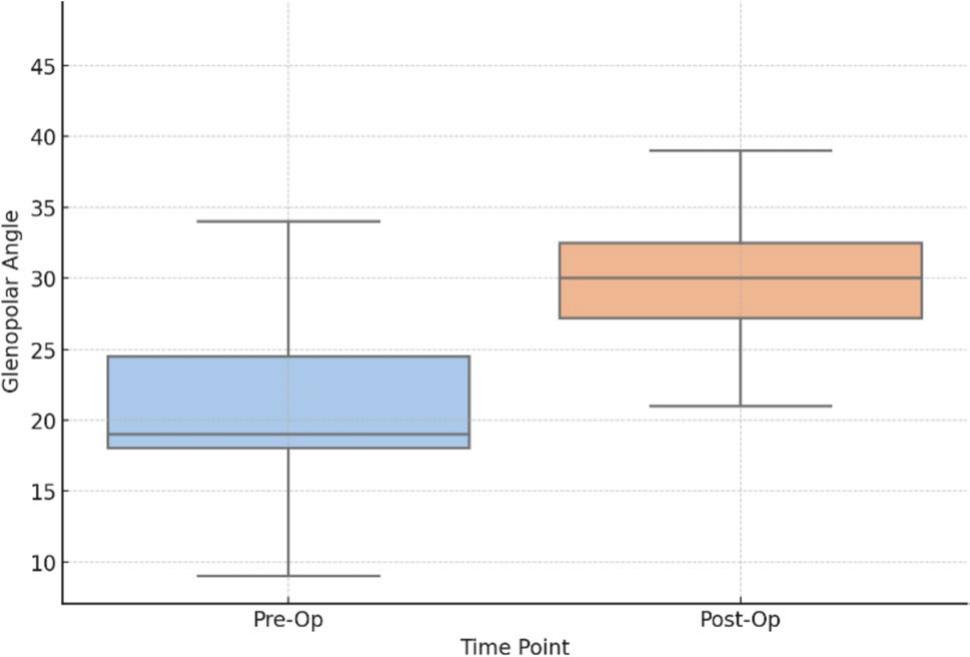
Comparison of the preoperative and postoperative glenopolar angle. Boxplot comparing the preoperative and postoperative glenopolar angle. A statistically significant difference was observed (*p* = 0.0008)

Regarding radiographic parameters, the postoperative glenopolar angle had a median value of 30° (IQR 25–33), while the preoperative glenopolar angle showed a median of 19° (IQR 18–25). There is a statistically significant difference between these values. Figure 5 shows the comparison of the glenopolar angle preoperative and postoperative. Postoperative medialization of the lateral border was minimal (median: 0 mm), whereas preoperative medialization of the lateral border had a median of 23 mm (IQR 20–30). There is a statistically significant difference between these values, with a *p* value =  < 0.0001.

No significant correlation was found between patient age and the Constant score (*p* = 0.8844). Additionally, no statistically significant difference in the Constant score of the affected shoulder was observed when comparing patients aged above and below 40 years (*p* = 0.8270). Although female patients exhibited a slightly higher median score, there was no statistically significant difference in Constant scores between male and female patients within this sample (*p* = 0.763). There was no statistically significant difference in functional outcomes or surgical timing between patients with and without additional injuries. The mean Constant Score of the affected shoulder was slightly higher in patients with associated injuries compared to those without (Supplementary Table 1), although this difference was not statistically significant (*p* = 0.514). Similarly, the time from trauma to surgery did not differ between groups (7.7 ± 4.0 days vs. 7.8 ± 2.6 days, respectively; *p* = 0.970).

**Fig. 6:**
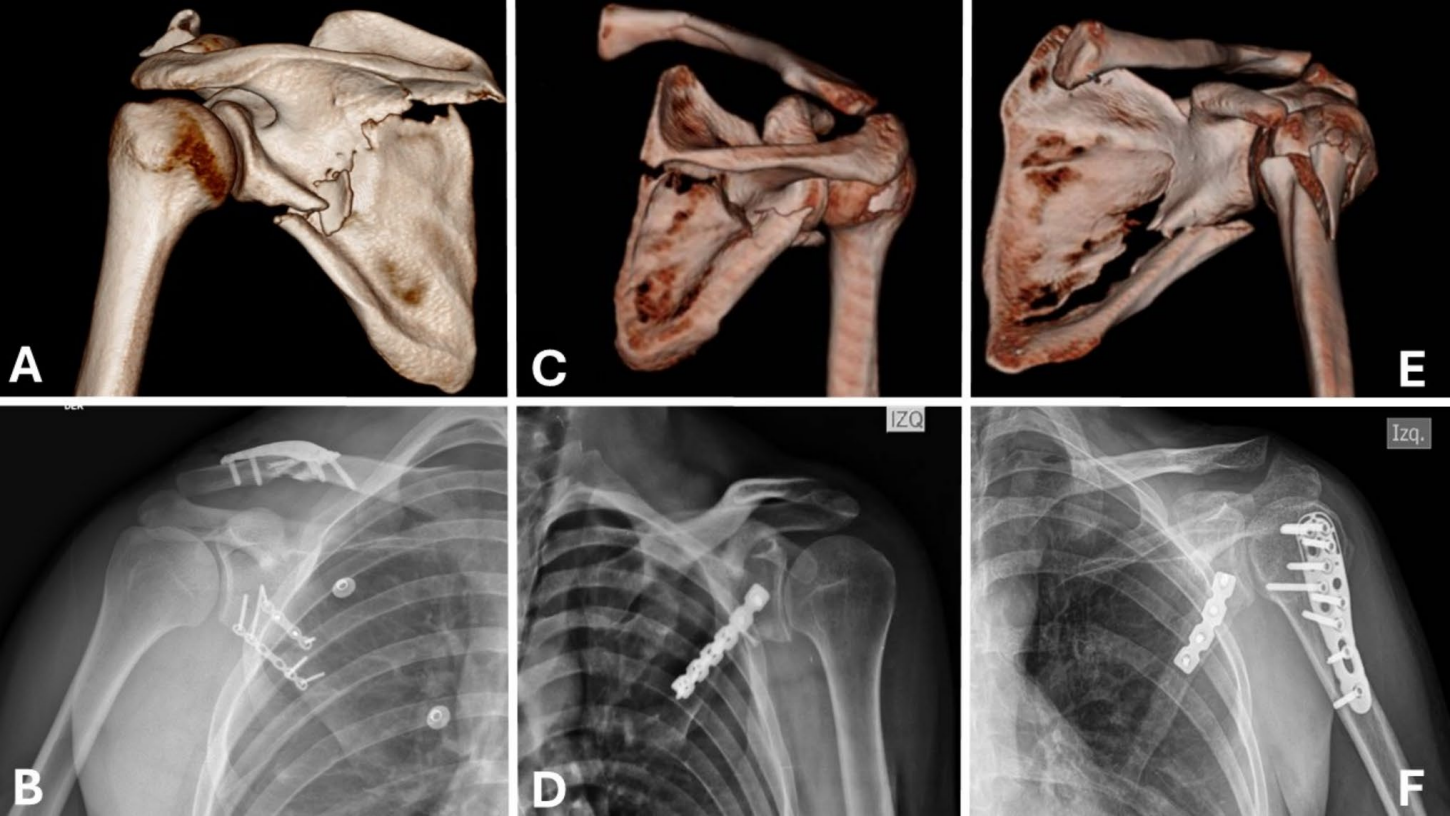
Radiological images showing the pre-and postoperative period of some patients. **A** Three-dimensional (3D) reconstruction CT scan showing a 14-B1 scapula fracture with a midshaft clavicle fracture in the right shoulder. **B** AP view of the shoulder 24 h postoperatively of Fig. 3A. **C** CT scan 3D reconstruction demonstrating a 14-B1 fracture of the left shoulder. **D** AP view of the shoulder 24 h postoperatively of Fig. 3C. **E** CT scan 3D reconstruction demonstrating a 14-B1 fracture with a proximal humerus fracture in the left shoulder. **F** AP view of the shoulder 24 h postoperatively of Fig. 3E

According to the AO/OTA classification, 11 patients (68.7%) had a 14-B1 extra-articular fracture and the remaining 5 had a 14-B2 fracture. No patients had reoperation or complications during follow-up, and all showed adequate reduction (Fig. 6) and there was a 100% union rate at the time of the final follow-up.

A multiple linear regression analysis was performed to identify predictors of functional outcomes, measured by the Constant score at 48 months postoperatively. The variables included in the model were: glenopolar angle (both preoperative and postoperative), medialization of the lateral humeral border (both preoperative and postoperative), patient age at the time of surgery, and the Constant score of the contralateral (non-operated) shoulder. Patients with incomplete data were excluded to maintain the robustness and validity of the analysis.

The model was statistically significant overall (*R*^2^ = 0.848, *p* = 0.027), explaining 84.8% of the variance in the Constant score. Among the predictors, only the contralateral Constant score showed a statistically significant association (*β* = 1.52, *p* = 0.001), suggesting that each additional point in the contralateral shoulder is associated with a 1.52-point increase in the operated shoulder’s Constant score. Other variables, including glenopolar angles, medialization of the lateral border, and age, did not reach statistical significance, see Table 1.

**Table 1:** Coefficients from the multiple linear regression model

Variable	Coefficient (*β*)	Standard error	95% CI (Lower)	95% CI (Upper)	*p* value
GPA post-Op	0.062	0.536	− 0.988	1.112	0.912
GPA pre-Op	0.009	0.247	− 0.475	0.492	0.973
Medialization post-Op	4.724	11.238	− 17.301	26.750	0.689
Medialization pre-Op	4.735	3.212	− 1.560	11.030	0.191
Age at surgery	0.290	0.224	− 0.258	0.838	0.243
Contralateral constant score	1.520	0.276	0.846	2.195	< 0.001

GPA, Glenopolar angle; Post-Op, Postoperative; Pre-Op: Preoperative

## Discussion

This study highlights the value of the Brodsky posterior approach in managing displaced extra-articular scapular fractures, showing excellent long-term functional outcomes and a low complication rate. Notably, contralateral shoulder function emerged as the only independent predictor of postoperative recovery, while radiographic correction parameters such as glenopolar angle or lateral border medialization did not show significant predictive value. These findings suggest that baseline shoulder capacity and overall functional status may play a more decisive role in recovery than anatomical alignment alone. This aligns with the growing emphasis on individualized patient assessment and supports the use of posterior approaches that preserve soft tissue integrity.

Our results are consistent with prior studies reporting favorable outcomes following surgical stabilization of scapular fractures. Jaikish and Sambandam (2020) described good or excellent results in 91% of patients treated with a modified Judet approach, with a mean Constant score of 80 [16]. In comparison, our cohort achieved higher scores, which may be attributed to the deltoid- and external rotator-sparing nature of the Brodsky approach. Similarly, Porcellini et al. (2019) found that minimizing infraspinatus detachment preserved muscle mass and external rotation strength [17]. Fandridis et al. (2018) also demonstrated excellent results using a posterior subdeltoid and external rotator–preserving approach, achieving full range of motion recovery and no muscle atrophy, supporting the idea that muscle-sparing techniques optimize long-term outcomes [15].

Although a statistically significant difference was observed between the Constant scores of the injured and contralateral shoulders (median difference: 2 points; *p* = 0.002), this difference is unlikely to be clinically relevant. In the absence of a scapular-specific MCID, we referenced values for proximal humerus fractures, estimated between 5.4 and 11.6 points [18]. Thus, the observed difference in our cohort likely falls below the threshold of perceptible clinical improvement. Most patients recovered near-normal shoulder function, reinforcing the efficacy of the Brodsky approach in restoring function.

Interestingly, although radiographic correction was consistently achieved, variables such as glenopolar angle and medialization did not significantly correlate with functional outcomes in the regression analysis. This is in line with previous literature, suggesting that radiographic realignment does not always translate into improved function, particularly when musculoskeletal integrity is preserved. Schroder et al. (2016) similarly found that functional results were more strongly influenced by range of motion and bilateral shoulder strength than by imaging parameters [19]. Our findings support this notion and highlight the importance of evaluating the contralateral shoulder during preoperative planning. In fact, the only independent predictor in our model was the Constant score of the contralateral limb (*β* = 1.52, *p* = 0.001), reinforcing the concept that pre-injury capacity and neuromuscular conditioning are essential determinants of outcome [20].

Although the initial hypothesis assumed that improved radiographic parameters would predict better postoperative function, our results suggest otherwise. Contrary to expectations, anatomical correction alone did not correlate with functional outcome, and baseline function of the uninjured shoulder proved more relevant. This underscores the need to interpret imaging improvements within a broader functional and patient-centered context.

This study has several limitations. The relatively small sample size limits the statistical power and generalizability of the findings. The absence of a nonoperative control group or comparison with other surgical approaches precludes definitive conclusions regarding the superiority of the Brodsky technique. Although the follow-up duration of up to 48 months is substantial, it may not fully capture late-onset complications such as post-traumatic arthritis or hardware-related issues. Functional outcomes were assessed exclusively using the Constant score, a validated and widely accepted instrument; however, it may not fully reflect patient satisfaction or quality of life. Although shoulder range of motion was thoroughly documented, external rotation strength was not consistently measured and thus could not be analyzed. This represents a limitation when assessing the protective effect of the Brodsky approach on the suprascapular nerve. Nonetheless, the excellent range of motion achieved, particularly in external rotation, provides indirect evidence of nerve integrity. Furthermore, patient occupation was not consistently recorded in medical records and could not be analyzed, despite its potential relevance to postoperative functional demands and return-to-work outcomes.

Despite these limitations, the study presents several notable strengths. All procedures were performed by a fellowship-trained shoulder specialist at a high-volume trauma center, ensuring consistency in surgical technique and postoperative care. The standardized use of the Brodsky posterior approach across the cohort minimizes variability and strengthens internal validity. The long-term follow-up allowed for meaningful assessment of functional and radiographic outcomes. Objective pre- and postoperative radiographic measurements, combined with multiple linear regression modeling, provided a comprehensive evaluation of factors influencing recovery. Additionally, the inclusion of range-of-motion data and contralateral shoulder comparisons support the functional relevance of the findings. Collectively, these methodological strengths enhance the reliability and clinical applicability of our conclusions.

In summary, the Brodsky approach offers a safe, effective, and anatomically respectful technique for the surgical treatment of displaced extra-articular scapular fractures. Its ability to preserve posterior soft tissue structures likely contributes to the favorable functional outcomes observed. While radiographic alignment remains important for surgical planning, our results suggest that baseline contralateral shoulder function is the most reliable predictor of postoperative recovery. These findings highlight the value of bilateral functional assessment in guiding surgical decision-making and rehabilitation strategies. Further prospective studies with larger cohorts and patient-reported outcomes are warranted to validate these results and expand on their clinical implications.

## Supplementary Information

Below is the link to the electronic supplementary material.

Supplementary file1 (XLSX 12 KB)

## Data Availability

No datasets were generated or analyzed during the current study.
